# A relative increase in circulating platelets following chemoradiation predicts for poor survival of patients with glioblastoma

**DOI:** 10.18632/oncotarget.21799

**Published:** 2017-10-12

**Authors:** Keeratikarn Boonyawan, Kenneth R. Hess, Jie Yang, Lihong Long, Qianghu Wang, Ravesanker Ezhilarasan, Alessandra Auia, Kristin D. Alfaro-Munoz, John F. de Groot, Krishna P. Bhat, Erik P. Sulman

**Affiliations:** ^1^ Department of Radiation Oncology, Ramathibodi Hospital, Mahidol University, Bangkok, Thailand; ^2^ Department of Biostatistics, The University of Texas MD Anderson Cancer Center, Houston, Texas, USA; ^3^ Department of Radiation Oncology, The University of Texas MD Anderson Cancer Center, Houston, Texas, USA; ^4^ Department of Translational Molecular Pathology and Neurosurgery, The University of Texas MD Anderson Cancer Center, Houston, Texas, USA; ^5^ Department of Neuro-Oncology, The University of Texas MD Anderson Cancer Center, Houston, Texas, USA

**Keywords:** glioblastoma, platelets, thrombocytosis, prognosis, survival

## Abstract

**Background:**

Thrombocytosis is triggered by and promotes tumor growth. The relationship between the change in circulating platelets after chemoradiation therapy (CRT) or adjuvant temozolomide (TMZ) and survival in glioblastoma remains unclear. We hypothesized that an increase in platelets after these treatments would be predictive of a shorter survival.

**Methods:**

We retrospectively reviewed data on 122 patients with newly diagnosed, pathologically proven glioblastoma who had been treated with surgery, followed by CRT and adjuvant TMZ, from 2007 to 2016. The association between the changes in blood count levels and survival was analyzed by the log-rank test. To adjust for confounding, we performed a multivariate analysis using known prognostic co-variates.

**Results:**

Patients were dichotomized on the basis of the relative change in platelets after CRT from the baseline: ≤30% increase, low (*n* = 101) *vs* >30% increase, high (*n* = 12). The median survival for high *vs*. low platelets were 11 *vs* 28 months (*p* = 0.0062). No significant survival differences were observed on the basis of platelet changes during adjuvant TMZ. Similarly, changes in lymphocyte counts were not significantly prognostic. On multivariate analysis, MGMT, performance status, and an increase in platelets after CRT were significantly associated with survival (HR for platelets, 4.5; 95% confidence interval, 1.6-12.6).

**Conclusions:**

Increased platelet counts after CRT are predictive of poor survival in glioblastoma. The effect is platelet specific and does not reflect bone marrow changes, as lymphocyte changes were not significantly prognostic. These results suggest an interaction between platelets and tumor aggressiveness. Thus, platelets serve as a novel, minimally invasive liquid biopsy for predicting outcome.

## INTRODUCTION

Thrombocytosis is associated with poor survival in various malignant tumors, including malignant mesothelioma, melanoma, glioblastoma, and breast, lung, esophageal, gastric, colorectal, renal transitional cell, endometrial, and ovarian cancers [1]. The mechanism of tumor-induced thrombocytosis has been extensively studied. The production of thrombopoietic factors from growing tumors, such as interleukin 1 (IL-1), IL-3, IL-6, IL-11, leukemia inhibitory factor, KitL, and oncostatin M, may be involved in thrombocytosis [1]. For instance, IL-6 is elevated in a variety of malignant tumors, including gastrointestinal cancer, renal cell carcinoma, prostate cancer, epithelial ovarian cancer, lung cancer, Kaposi's sarcoma, and glioblastoma [2]. Moreover, an increasing level of IL-6 is significantly associated with thrombocytosis, and blocking IL-6 leads to progression-free survival and overall survival benefits in patients with ovarian cancer [3].

Platelets promote tumors in two phases: intravascular and extravascular. In the intravascular phase, platelets release immunoregulatory molecules such as PDGF, TGF-, and IFN-g to decrease the reactivity of NK cells [4, 5]. The expression of MHC class I on the platelet surface while binding to tumor cells causes NK cells to no longer recognize tumors [6]. In addition, platelet-derived VEGF suppresses the development of dendritic cells and antigen-presenting cells into maturity [7]. This mechanism leads to circulating tumor cells surviving the immune system.

In the extravascular phase, platelets facilitate tumor aggressiveness and metastasis by several mechanisms: (i) platelets activated by shear stress lead to tumor cell attachment to the subendothelial extracellular matrix *in vitro* [8]; (ii) tumor-platelet interaction stimulates VEGF and tissue factors, promoting tumor adhesion and vessel hyperpermeability [9, 10]; and (iii) local embolization of the microvasculature with tumor-platelet aggregation encourages extravasation [11].

Not only do platelets promote tumor metastasis, they stimulate tumor angiogenesis by releasing proangiogenic factors such as VEGF and PDGF at higher levels than antiangiogenic factors [12, 13]. VEGF is the most powerful positive regulator of angiogenesis, and its level is twice as high in cancer patients as in healthy controls [14, 15]; PDGF has been associated with tumor growth and angiogenesis in many studies [16–19]. Podoplanin, a powerful inducer of platelet aggregation, binds with its receptor, C-type lectin receptor, on the tumor surface to induce cancer growth and metastasis [20]. Recent studies have shown that this interaction between podoplanin and C-type lectin receptor is a promising target for cancer therapy [21].

Communication between tumors and platelets has been recently described. Specifically, tumor-educated platelets receive RNA from tumor cells that may support tumor growth and metastasis. Best et al. reported the differences between the platelet-mRNA sequence in cancer patients and healthy individuals [22]. These results indicated that tumor-educated platelets serve as a novel blood-based biomarker of cancer.

Brockman et al. found that preoperative thrombocytosis was associated with poor survival in GBM patients [23]. However, the relationship between the change in circulating platelets and the survival duration of patients with GBM remains unclear. Moreover, platelets are readily obtained from the peripheral blood and represent a novel liquid biopsy. Tumor-educated platelets containing tumor-specific mRNA have been demonstrated for a variety of tumors, including GBM [22]. Thus, in this study, we determined the association between the change in platelet count after concurrent chemoradiation therapy (CRT) or adjuvant temozolomide (TMZ) and the survival duration of GBM patients. We found that an increased platelet count after CRT was a poor prognostic factor in these patients. The results of this study support future biomarker studies to identify the platelet-mRNA profile in patients who have an increased platelet count after CRT and to develop new targeted therapies.

## MATERIALS AND METHODS

In this retrospective study, we reviewed clinical and laboratory records to identify patients with GBM who had been treated in the Department of Radiation Oncology at The University of Texas MD Anderson Cancer Center (Houston, Texas) from January 2007 to December 2016. The inclusion criteria were patients who were newly diagnosed, had pathologically proven GBM, and had undergone surgery, followed by CRT and adjuvant TMZ. Patients who had undergone adjuvant treatment at another institution and those with potential confounders for thrombocytosis, including infection, thrombosis, underlying hematologic disease, and anti-platelet medication, were excluded. The number of patients in the population before the exclusion criteria were applied was 144, and 23 patients were excluded. One hundred twenty-two patients were enrolled in the study.

**Figure 1:**
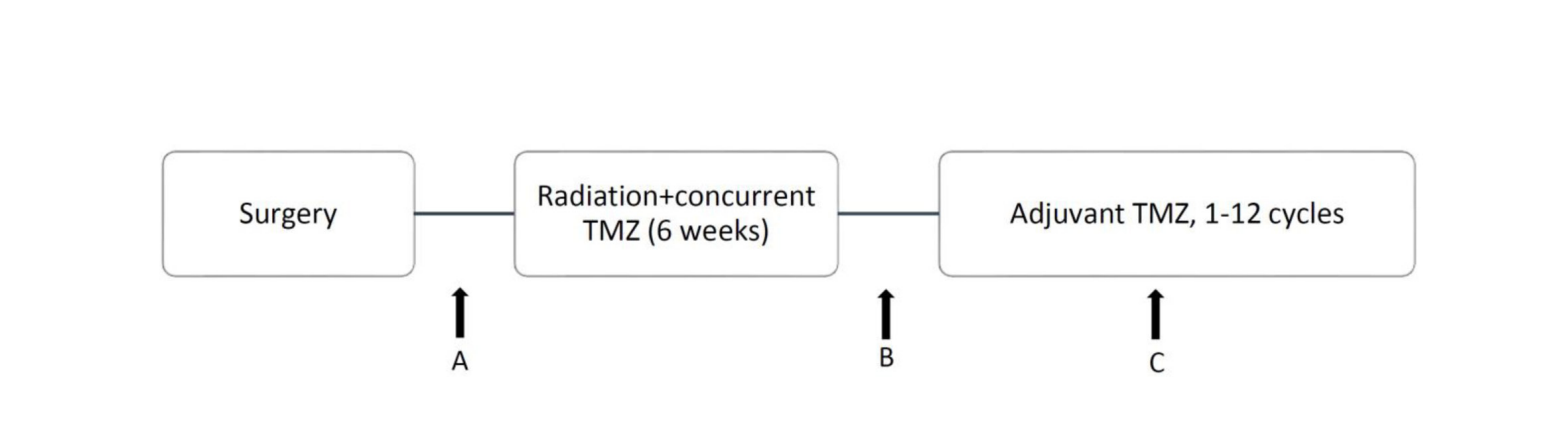
Timing of treatment and calculation points of platelet and lymphocyte counts: A, B, and C represent platelet and lymphocyte counts before CRT, after CRT, and during adjuvant TMZ, respectively

Patients’ platelet counts had been collected before CRT, after CRT, before adjuvant TMZ, and during adjuvant TMZ. To determine whether the change in platelets was affected by bone marrow response, we recorded the lymphocyte count as a reference marker, along with the platelet counts [24] (Figure 1). We measured the changes in platelet and lymphocyte counts during CRT and adjuvant TMZ. The CRT phase was calculated on the basis of the difference in blood counts from points A and B, and the adjuvant TMZ phase was calculated on the basis of the difference from points B and C (Figure 1). Blood counts before CRT (point A) were collected at the beginning of RT or within 2 weeks before RT. The blood count after CRT (point B) was defined by the date of RT completion or within 2 weeks after RT, and blood counts during adjuvant TMZ (point C) were collected in the middle of adjuvant TMZ. To avoid confounding errors due to recurrent or progressive disease, we measured the blood counts during adjuvant TMZ (point C) instead of those at the end of TMZ. Patients without available blood test data after treatment were excluded from the analyses. In addition, patient age, sex, Karnofsky performance status (KPS), IDH status, O^6^ methylguanine DNA methyltransferase gene (*MGMT*) promoter methylation status, extent of resection (gross total or subtotal), gross tumor volume before RT (GTV), CRT dose, and adjuvant TMZ cycles completed were included as baseline patient characteristics and treatment factors.

To define the optimal magnitude of the change in platelets, patients were dichotomized on the basis of the relative change in platelets from the CRT and adjuvant TMZ phases. A threshold of 30% relative change was selected to optimize the difference between groups. A ≤30% increase in platelets was defined as “low platelets,” and a >30% increase was defined as “high platelets.” Similarly, a >30% increase in the lymphocyte count was defined as “high lymphocytes” and a ≤30% increase as “low lymphocytes.”

Radiation was given to a total dose of 60 Gy to gross disease and the post-operative resection cavity and 50 Gy to areas of microscopic tumor. Adjuvant TMZ was administered concurrently with RT at a dose of 75 mg/m^2^ per day, starting on the first day of RT and continuing until the last day. Four weeks after the completion of CRT, TMZ was given at 150-200 mg/m^2^ for 5 days per 28-day cycle, with the intention of being delivered for a total of 6-12 cycles.

Survival duration was measured from the starting date of CRT to the date of death or last follow-up to determine the CRT effect and from the starting date of adjuvant TMZ to the date of death or last follow-up to determine the adjuvant TMZ effect. The date of last follow-up was used for patients who were still alive. The association between changes in blood counts and patient survival was analyzed by the Kaplan-Meier method and the log-rank test; this method was used in all patients, and the subgroup was divided by *MGMT* methylation status. A multivariate Cox proportional hazards analysis was performed using known prognostic covariates, including patient age, performance status, MGMT methylation status, IDH mutation status, and extent of resection. The association between GTV and platelet count was analyzed by the Spearman rank correlation coefficient. A p value of <0.05 was considered significant for the analysis. All analyses were conducted using JMP statistical software and S+ version 8.2 for Windows (TIBCO Software, Inc.).

## RESULTS

### Patients and overall survival

The characteristics of the 122 cases are presented in Table 1. The median age of the patients was 57 years; 67% were male, and 91% had KPS >70. Most (72%) patients had IDH wild-type tumors, and 58 (48%) had *MGMT* promoter methylation. More than 70% of patients underwent gross total resection, and the mean GTV for RT planning was 53.9 cm^3^. The majority of patients (53%) had undergone 6-12 cycles of adjuvant TMZ; the reasons for early termination of TMZ (less than 6 cycles) were toxicity, disease progression, loss to follow-up, and death. The median survival duration of all patients was 26 months (95% CI, 20-37 months). The 1-year and 2-year overall survival rates were 84% and 54%, respectively (Table 2).

**Table 1:** Characteristics of GBM patients

Parameter	All patients, n (%)^1^
Median age (years)	57
<50	89 (73)
≥50	33 (27)
KPS	
≤70	11 (9)
>70	111 (91)
MGMT status	
Methylation	58 (48)
Unmethylation	51 (42)
Undetermined	8 (6)
NA	5 (4)
IDH1 status	
Wild-type	88 (72)
Mutation	10 (8)
NA	24 (20)
Extent of resection	
Gross total	89 (73)
Subtotal or biopsy	33 (27)
Median GTV (cm^3^)	53.9
Adjuvant TMZ cycle	
Only concurrent	8 (7)
<6	45 (37)
6-12	53 (43)
Ongoing	11 (9)
NA	5 (4)

^1^Except where indicated.

**Table 2:** Survival between low and high blood counts after 2 treatment phases

Patients	CRT			Adjuvant TMZ		
	Median survival duration (months)	p value	HR	Median survival duration (months)	p value	HR
Platelet analysis						
High platelets	11	0.0062	3.4 (1.6-7.5)	22	0.14	1.8 (0.9-3.8)
Low platelets	28			17		
Lymphocyte analysis						
High lymphocytes	18	0.43	1.6 (0.6-4.4)	22	0.16	1.6 (0.9-2.9)
Low lymphocytes	26			18		

### Platelet analysis

#### Concurrent CRT

**Figure 2:**
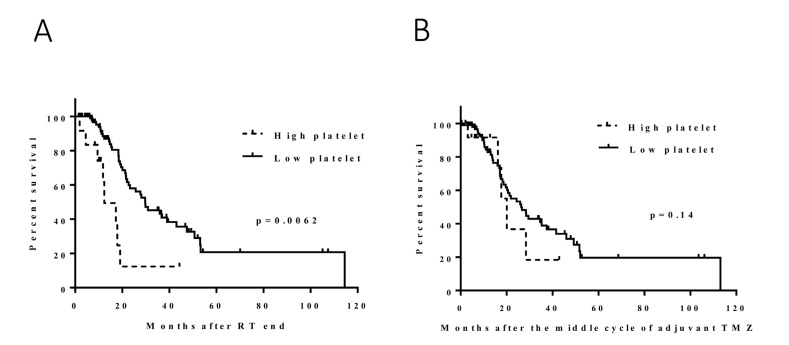
Effect of the change in platelets following CRT (**A**): the high-platelet group had significantly shorter survival durations than did the low-platelet group (median, 11 and 28 months; p = 0.0062). Effect of the change in platelets during adjuvant TMZ (**B**): no significant survival difference was found between the high and low platelet groups (median, 22 and 17 months; *p* = 0.1).

**Figure 3:**
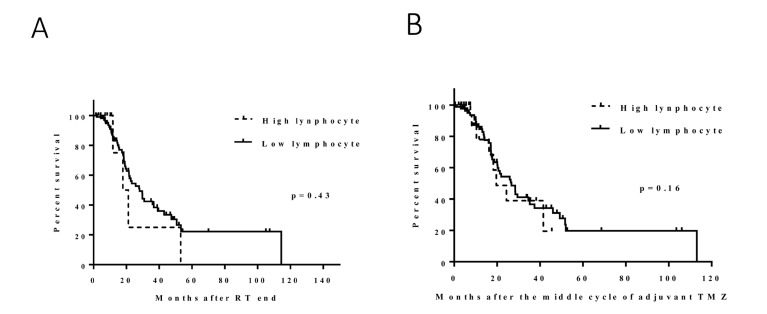
Effect of the change in lymphocytes following CRT (**A**): no significant survival difference was found between the high and low lymphocyte groups (median, 18 and 26 months, p = 0.43). Effect of the change in lymphocytes during adjuvant TMZ (**B**): no significant survival difference was found between the high and low lymphocyte groups (median, 16 and 17 months, *p* = 0.16).

We compared hematologic data before and after CRT (points A and B in Figure 1); the median change in platelets was -29 k/μl (range, -387 to 179 k/μl). The high-platelet group (*N* = 12) had significantly shorter survival durations than did the low-platelet group (*N* = 101) (median, 11 and 28 months, respectively; hazard ratio [HR], 3.4 [1.6-7.5]; *p* = 0.0062) (Figure 2). The subgroup analysis by MGMT methylation status included 100 patients for whom an *MGMT* methylation profile was available. Fifty-eight patients had MGMT methylation (7 patients with high platelets, 47 patients with low platelets, and 4 patients with missing data). The survival duration was considerably shorter in the high-platelet group (median, 11 months versus 37 months; HR, 3.7 [1.2-11.6]; *p* = 0.047), which was consistent with the results for all patients (Figure 3). The group with unmethylated *MGMT* was not analyzed because the sample size of the high-platelet group was too small (*N* = 3).

#### Adjuvant TMZ

Similar to what was done for the platelet counts, we compared platelet counts before and during adjuvant TMZ (points B and C in Figure 1) and determined that the median change in platelets was -8 k/μl (range, -295 to 524 k/μl). No obvious survival difference was found between the high (*N* = 21) and low (*N* = 88) platelet groups. The median survival durations were 22 and 17 months, respectively (HR, 1.8 [0.9-3.8]; *p* = 0.14) (Figure 2). In the *MGMT* methylated group, no significant correlation was noted between the change in platelets and survival (median, 29 [*N* = 11] and 36 [*N* = 40] months, respectively; HR, 1.6 [0.5-5.7]; *p* = 0.49) (Figure 2). The number of patients with high platelets and unmethylated *MGMT* was too small for analysis.

We performed a multivariable analysis of survival to adjust for known prognostic covariates on the prognostic significance of platelet changes. Our findings revealed that *MGMT* status, KPS, and an increase in platelets after CRT were significantly associated with survival (HR for platelets, 4.5; 95% CI, 1.6-12.6). In our cohort, patient age, IDH mutational status, and extent of resection were not significantly associated with survival (Table 3).

**Table 3:** Multivariate Cox proportional hazards analysis to adjust for confounding

Covariate	HR (95% CI)	p value
Change in platelets after CRT		
High	4.5 (1.6-12.6)	0.0039
Low	reference	
Age, years		
>50	1.0 (0.4-2.4)	0.93
≤50	reference	
KPS		
>70	0.1 (0.02-0.3)	0.0001
≤70	reference	
IDH1 status		
Mutation	0.2 (0.03-1.8)	0.17
Wild-type	reference	
MGMT status		
Methylation	0.4 (0.2-1.0)	0.042
Unmethylation	reference	
Extent of resection		
Gross total	1.4 (0.6-3.2)	0.49
Subtotal or biopsy	reference	

We considered the possibility that the tumor burden was directly correlated with the platelet numbers and therefore correlated GTV to platelet counts before CRT or the change before and after CRT. We found that there was little to no correlation between GTV and either platelet count before CRT or the change in platelet count after CRT (Spearman rank correlation coefficient, 0.06 [95% CI, -0.14-0.26] and 0.01 [95% CI, -0.19-0.22]).

### Lymphocyte analysis

To determine whether the observed change in platelets was a general phenomenon of bone marrow effects, we compared lymphocyte counts before and after CRT (points A and B in Figure 1) and before and after TMZ (points B and C in Figure 1). The median change in lymphocytes before and after CRT was -0.7 k/μl (range, -5.7 to 1.3 k/μl). This change in lymphocyte count was not associated with survival: the median survival durations in the high (*N* = 10) and low (*N* = 103) lymphocyte count groups were 18 months and 26 months, respectively (HR, 1.6 [0.6-4.4]; *p* = 0.43) (Figure 3A). The median change in lymphocytes before and during TMZ was -0.05 k/μl (range, -1.35 to 2.16 k/μl). No significant survival difference was found between the high (*N* = 32) and low (*N* = 77) lymphocyte groups (median, 16 and 17 months, respectively; HR, 1.6 [0.9-2.9]; *p* = 0.16) (Figure 3B).

## DISCUSSION

Increased platelet counts after CRT are predictive of poor survival in patients with GBM. This effect is platelet specific and does not reflect general bone marrow changes, as lymphocyte changes were not significantly associated with survival duration in our study. Our results suggest that an interaction exists between platelet counts and tumor aggressiveness.

The findings in this study indicate that a change in platelet count is a prognostic marker for GBM, as the survival duration of patients with high platelet counts after CRT was significantly shorter than was that of patients with low platelets. Our data are consistent with those of a study by Brockman et al. [23], who investigated the association between platelet count and GBM prognosis and found that preoperative thrombocytosis was predictive of poor survival. Ingo et al. [25] found that thrombocytosis was associated with tumor burden: the platelet counts gradually increased, peaking at the time of surgery.

The studies by Brockman et al. and Ingo et al. focused on platelet counts before surgery. In our study, we determined the change in platelets during adjuvant treatment. Even though the gross tumor had been removed, CRT played a major role in removing microscopic residual disease and providing locoregional control. After CRT, platelet counts were reduced in most patients; high platelet counts might have reflected resistance to CRT, and these patients likely had more microscopic residual tumor. Consequently, the high-platelet group experienced shorter survival durations. Another difference between our studies and others is that we evaluated the change in platelets over time instead of at a particular point in treatment to account for variations in baseline platelet counts. This method allows patients with low baseline platelet counts who experienced an increase after CRT to be included in the high platelet count group despite not meeting the criteria for thrombocytosis (>400 k/ml).

These findings also confirm those of a report by Williams et al. [24], who found that a reduction in platelet count during CRT is predictive of prolonged survival. They measured the change in platelets at weeks 1 and 6 of a radiation therapy course and found that patients who experienced an increase in platelets had a shorter survival duration than did patients who did not; the median survival durations were 11.8 months versus 19.6 months, respectively. In our study, we not only investigated the change in platelets after CRT but also during adjuvant TMZ and found that the change during adjuvant TMZ was not correlated with survival duration. It is possible that after completing CRT, some patients experienced a reduction in platelet count to lower than the normal baseline; therefore, after they experienced an increase of >30%, their counts still did not exceed the upper normal limit. Consequently, the survival durations between the low- and high-platelet count groups during adjuvant TMZ were not significantly different.

Our results provide additional information with regard to molecular profiles, such as *MGMT* methylation and IDH mutation status. GBM patients with *MGMT* methylation have been found to have significantly longer survival durations than those with unmethylated *MGMT* [26]. Likewise, in this study, we found that *MGMT* methylation status was a significant prognostic factor on multivariate analysis (*p* = 0.042). In addition, MGMT methylation patients with high platelet counts after CRT had remarkably shorter survival durations than did those with low platelet counts (median, 11 months versus 37 months, respectively; *p* = 0.047).

In the multivariate analysis, the increases in platelet count, KPS, and MGMT methylation status were associated with survival, which is consistent with the published data [26]. However, the extent of resection was not a prognostic factor in this study. This might be because 33% of the subtotal resection group underwent re-resection after completing CRT, which diluted the effect of gross total resection on survival. The GTV analysis revealed no association between GTV volume and the platelet count before CRT or the change in platelet count after CRT. These findings may be explained by the nature of GBM; as it is a highly infiltrative disease, the GTV contoured by T1-enhanced MRI did not include all microscopic residual disease.

The major limitation of our study is its retrospective nature, which may have led to a higher percentage (20%) of IDH mutant patients, who had better survival outcomes (median survival, 26 months) than did those in previous publications [27]. The 30% cutpoint used to distinguish our platelet survival groups has not been validated in an independent cohort. Some patients were excluded because of a lack of hematologic documentation; however, on the basis of the available data, the relationship between the change in platelets and survival duration was clearly demonstrated. According to the use of lymphocyte counts by Williams et al., we recorded >30% versus ≤30% changes in lymphocytes counts and found that the change in lymphocytes was not associated with survival after CRT or adjuvant TMZ. Thus, the prognostic power of the change in platelets was not associated with a bone marrow response.

The tumor-platelet interaction has been investigated in several studies. Buergy et al. [1] reported that growing tumors release cytokines that contribute to the increase in platelets. Meanwhile, many researchers have demonstrated mechanisms by which tumors are promoted by platelets [3–18]. These results support our findings that clinical outcome is associated with platelet counts in GBM patients. More importantly, the present study provides updated molecular profiles and used modern treatment techniques, including image-guided neurosurgery and intensity-modulated radiation therapy. Therefore, the effect of the change in platelets is promising as a novel prognostic factor.

Our study revealed the interaction between platelets and the aggressiveness of GBM. An increase in platelets during CRT is a poor prognostic factor and patients may require closer follow-up or more intensive treatment. In addition, a liquid biopsy using tumor-educated platelets is a minimally invasive procedure for real-time disease monitoring. A combination of increased platelet data and signatures from tumor-educated platelets may serve as a prognostic or predictive marker for patients with GBM.
